# Comparison of Nutritional Compositions and Essential Oil Profiles of Different Parts of a Dill and Two Fennel Cultivars

**DOI:** 10.3390/foods10081784

**Published:** 2021-07-31

**Authors:** Yuanpeng Hao, Jiamu Kang, Xiaoqi Guo, Rui Yang, Yuliang Chen, Jingyi Li, Lei Shi

**Affiliations:** 1 Key Laboratory of Plant Resources and Beijing Botanical Garden, Institute of Botany, Chinese Academy of Sciences, Beijing 100093, China; yphao@ibcas.ac.cn (Y.H.); guoxiaoqi@ibcas.ac.cn (X.G.); yangrui1993@ibcas.ac.cn (R.Y.); jingyileesd@126.com (J.L.); 2 University of Chinese Academy of Sciences, Beijing 100049, China; 3 College of Food Science & Nutritional Engineering, China Agricultural University, Beijing 100083, China; Jiamu_kang@163.com; 4 Institute of Biotechnology, Gansu Academy of Agricultural Sciences, Lanzhou 730070, China; chenyl925@163.com

**Keywords:** Apiaceae plants, plant parts, efficient utilization, multivariate analysis, food additives

## Abstract

Fennel and dill are widely used as food additives owing to their various biological activities, such as antioxidants, antimicrobials, food-preservatives, and seasoning capacities. Herein, the nutritional composition and essential oil (EO) chemical profiles of fruits, umbels, stalks, and roots from one dill and two fennel cultivars were evaluated. The fruits had the highest content of crude protein (≥15%), crude fat (≥8%), and phosphorus (≥0.5%), and exhibited the highest total energy (≥20 MJ/kg) and EO yield (≥2%). Moreover, estragole (86.56% in Fdf), anethole (71.17% in Fhf), fenchone (16.74% in Fhf), limonene (50.19% in Agf), and carvone (42.41% in Agf) were the main components of the EOs generated from the fruits. The chemical profiles of EOs in the roots were significantly different from those of the aerial parts of the fennel and dill; thus, the roots and aerial parts could be distinguished based on myristicin (Variable Importance in Projection (VIP) = 1.90399) and apiol (VIP = 1.85922). The EO components of the aerial parts varied remarkably, and the chemical markers for differentiating these three cultivars were anethole (VIP = 1.36571), estragole (VIP = 1.30292), and carvone (VIP = 1.11947). Overall, our results provide a noteworthy chemical basis for further development of fennel and dill, especially as food additives.

## 1. Introduction

The Apiaceae plant family comprises some of the most important medicinal and aromatic plants cultivated worldwide [1]. Fennel (*Foeniculum vulgare* Mill.) and dill (*Anethum graveolens* L.) are the most vital medicinal and aromatic plants in the Apiaceae family [2], as they are rich in fatty and amino acids, fiber, minerals, vitamins, flavonoids, phenols, and volatile components. Consequently, fennel and dill are useful for obtaining stomachic, galactagogue, carminative, and mildly diuretic substances [3,4,5,6]. Moreover, they are industrially and economically valuable herbs [7,8].

Fennel and dill fruits can be eaten as a condiment because of their unique flavor and abundant nutritional profile [9]. Furthermore, fennel is an excellent plant-based source of potassium, sodium, phosphorus, and calcium [10]. The nutritional composition of different parts of such plants varies; for example, the mineral content of fennel leaves is higher than that of fennel fruits [11]. Minerals play an important role in animal reproduction and are vital for healthy growth. For instance, to meet the need for milk synthesis after childbirth, the demand for calcium increases during lactation. Many important enzymes also require trace minerals, which are vital for enzymatic and structural functions [12]. Therefore, fennel and dill by-products can also be used as feed additives to promote the healthy growth of animals, increase gastrointestinal (GI) peristalsis and intake, and improve intestinal microorganism balance [13,14].

Consumer preference for clean-label products requires the food industry to reformulate products by replacing artificial additives, such as colors, flavors, and preservatives, with natural alternatives [15]. Essential oils (EOs) are a complex mixture of secondary metabolites (terpenes, phenolic compounds, and alcohols) that can be extracted from several parts of medicinal and aromatic plants. EOs exhibit extensive biological activities, including antioxidant, antimicrobial, and anti-inflammatory activities [16]. In particular, fennel and dill EOs have a distinctive flavor and possess antibacterial and food preservation capabilities. Thus, there is substantial potential for the application of such oils in the food industry. Notably, these oils are generally recognized as safe (GRAS) substances and have the potential to be used as food additives for vegetables, fruits, baked goods, meat, and dairy products [17,18,19]. Certain EO components from fennel and dill are used in the food, biopesticide, and pharmaceutical industries. For instance, carvone was identified as an effective potato sprout inhibitor [20] that accounts for the flavor and antiflatulence characteristics of dill [8]. Furthermore, limonene possesses a pleasant lemon flavor and is used as an antibacterial additive in common foods (e.g., fruit juices, sweets, and ice cream), and it is easily digested and absorbed by the GI tract [21,22].

Many studies have focused on the biological activities of fennel and dill. However, a lack of systematic studies on fundamental chemical information currently limits the application of these plants. Thus, given the immense nutritional value and wide range of biological activities of fennel and dill, this study aimed to investigate the chemical diversity of different parts of the plants and cultivar species. The nutritional composition and EO chemical profiles of different parts of locally grown fennel and dill species were measured. Moreover, a multidimensional exploration regarding EOs was performed in this study. The detailed chemical characterization of different parts of fennel and dill plants will provide baseline data to guide their application as food additives.

## 2. Materials and Methods

### 2.1. Sample Collection and Preparation

Two fennel cultivars of *Foeniculum vulgare* “Dwarf” (Fd) and *Foeniculum vulgare* “High” (Fh), and one dill cultivar of *Anethum graveolens* (Ag), were collected from Minqin County in Gansu Province, where the climate is temperate, continental, and arid. The studied plants were grown on soft and sandy soils, and these cultivars were commercially available. Thirty individual samples of each plant were wholly (including roots) harvested at dawn between September and October 2018 and stored at room temperature (20–25 °C). Each plant was divided into four parts: fruit, umbel, stem, and root. Abbreviations corresponding to a total of twelve samples are as follows: Fd fruit (Fdf), Fd umbel (Fdu), Fd stem (Fds), Fd root (Fdr), Fh fruit (Fhf), Fh umbel (Fhu), Fh stem (Fhs), Fh root (Fhr), Ag fruit (Agf), Ag umbel (Agu), Ag stem (Ags), Ag root (Agr).

### 2.2. Determination of Nutritional Composition from Plant Materials

Dry matter, ash, phosphorus, and calcium contents were determined according to the AOAC procedures [23]. Acid detergent fiber (ADF) and neutral detergent fiber (NDF) were determined using heat-stable α-amylase and sodium sulfite [24]. The total energy was determined using a parr-6400 oxygen bomb calorimeter. A Kjeltec ^TM^ Sampler8420 Automatic Kjeldahl nitrogen analyzer (FOSS) was used to measure the crude protein content. Total fat was measured using an ANKOM^XT15^ automatic fat analyzer.

### 2.3. Extraction and Yield Determination of EOs

Air-dried samples were ground and subjected to steam distillation using a Clevenger-type apparatus for 3 h (100 g sample:1000 mL water). The isolated EOs were dried over anhydrous sodium sulfate and stored at 4 °C in an amber bottle before gas chromatography-mass spectrometry (GC-MS) analysis. The EO yields were estimated based on the dry weight of the plant materials (in % *v*/*w* for 100 g of dried raw material).

### 2.4. Gas Chromatography-Mass Spectrometry (GC-MS) Analysis

The EOs were filtered and diluted in n-hexane at a ratio of 1:200. These samples were then detected via GC-MS (7890A-7000B, Agilent Technologies, Santa Clara, CA, USA) analysis with an HP-5MS column (30 m × 250 μm × 0.25 μm; Agilent Technologies). The injector temperature was 250 °C. The oven program was conducted as follows: 1 μL of the sample was injected in a 100:1 split mode. The temperature was maintained at 40 °C for 2 min, and the linear ramp reached 152 °C at a rate of 4 °C·min^−1^; the second ramp attained 310 °C at a rate of 100 °C·min^−1^. The mass spectrometry conditions were set as follows: ionization energy, 70 eV; electronic impact ion source temperature, 230 °C; quadrupole temperature, 150 °C; and mass range, 40–700 u. The EO compounds were identified by comparing the National Institute of Standards and Technology (NIST) 17 library spectra and the retention index (RI) [25]. The RI values were determined using n-alkane (C7-C40) hydrocarbons under the same conditions. The chemical profiles of the EOs were obtained from the corresponding peak areas.

### 2.5. Statistical Analysis

Variance analysis was performed to evaluate the significance of differences using SPSS software (IBM SPSS 25.0; SPSS Inc., Chicago, IL, USA) and results with *p* values < 0.05 were considered significant. Heatmaps, Spearman’s rank correlation analysis (*p* < 0.05) results, UpSet plots, Venn diagrams, and principal component analysis (PCA) plots were obtained using the R platform. To further distinguish the differences and relationships among EOs, supervised statistical data treatment was performed using OPLS-DA by SIMCA (Version 14.1, Umetrics, Umea, Sweden).

## 3. Results and Discussion

### 3.1. Morphological Observations

**Figure 1:**
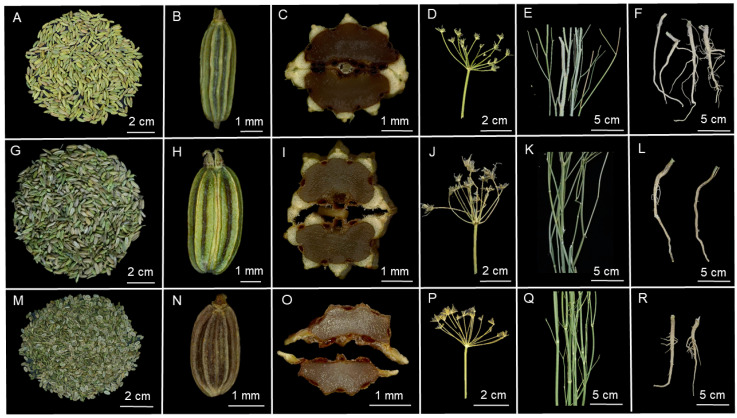
Representative images of different parts of one dill and two fennel cultivars. (**A**–**C**) *Foeniculum vulgare* “Dwarf” fruit (Fdf); (**D**) *F. vulgare* “Dwarf” umbel (Fdu); (**E**) *F. vulgare* “Dwarf” stalk (Fds); (**F**) *F. vulgare* “Dwarf” root (Fdr); (**G**–**I**) *F. vulgare* “High” fruit (Fdf); (**J**) *F. vulgare* “High” umbel (Fhu); (**K**) *F. vulgare* “High” stalk (Fhs); (**L**) *F. vulgare* “High” root (Fhr); (**M**–**O**) *Anethum graveolens* fruit (Agf); (**P**) *A. graveolens* umbel (Agu); (**Q**) *A. graveolens* stalk (Ags); (**R**) *A. graveolens* root (Agr).

As shown in Figure 1, the fruits of Fd and Fh were plump and shared a similar shape (ridged and oblong to ovoid), size (1.5–2.5 mm broad; 4–6 mm long), and coloring of the fruit epidermis (green). By contrast, the fruits of Ag were oblate, with a smaller size (1–2 mm broad; 2–4 mm long), and a brown epidermis. The fruits of Fd, Fh, and Ag were cremocarps, comprising a pair of symmetrical carpels (Figure 1C,I,O). The EOs were distributed in the oil ducts, which were oval or semicircular. Six oil ducts exist in the fixation locations of cremocarps. Upon detailed examination, we observed a side groove in each of the two strong ribs, and four oil ducts were located under the side groove, and two additional oil ducts were located on the ventral side. On short pedicels of each umbel section, 10–50 fruits were present (Figure 1D,J,P). The stalks of Fd, Fh, and Ag were smooth, bright green, cylindrical, and erect (Figure 1E,K,Q). The roots of Fd, Fh, and Ag also shared similar cylindrical shapes and sizes, with colors ranging from white to brown.

### 3.2. Analysis of Nutritional Compositions

**Figure 2:**
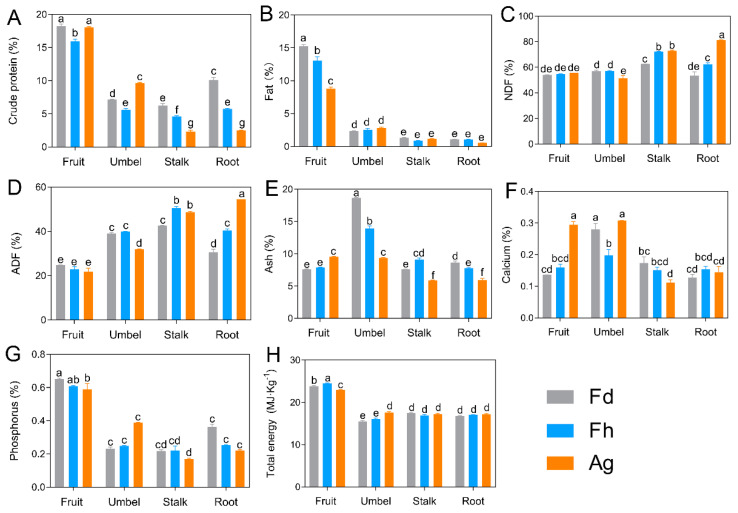
Nutritional composition (**A**–**G**) and total energy (**H**) of fruits, umbels, stalks, and roots from three Apiaceae species. Acid detergent fiber (ADF) and neutral detergent fiber (NDF); *Foeniculum vulgare* “Dwarf” (Fd), *F. vulgare* “High” (Fh), *Anethum graveolens* (Ag). Different letters indicate significant differences (*p* < 0.05).

Species-related factors and different parts of the plants had significant effects on the nutrient composition and total energy parameters (*p* < 0.05; Figure 2). The crude protein (15.98–16.22%) and fat contents (8.78–15.52%) of the fruits (Fdf, Fhf, and Agf) were higher than those of the umbels, stalks, and roots. Moreover, the fruits had the highest concentration of phosphorus (0.59–0.65%) and total energy values (22.97–24.50 MJ/kg); however, they had lower NDF, ADF, and ash contents. The level of phosphorus of fruits from dill and fennel observed in this study was higher than that of a report by a previous similar study (0.1745–0.3285%) [26]. The level of crude protein and ADF of Fdf and Fhf was consistent with the previous report from fennel fruits (15% crude protein and 24% ADF) [14]. Stalks and roots (except for Fdr) exhibited the highest NDF and ADF contents, followed by the umbels and fruits. The umbels contained more ash (9.66–18.66%) than the other parts. Total ash content could be an indicator of the total mineral content in the plant materials. In this context, it can be inferred that Fdu contained the highest amount of minerals. Agu contained the highest amount of calcium (0.31%), and the lowest amount was found in Ags (0.11%).

Plants are a good source of nutritional components, such as fiber, minerals, and proteins, for humans and other animals. According to the U.S. Department of Agriculture, fennels are rich in dietary fiber and vitamins, relative to human needs [18]. *Foeniculum vulgare* contains appreciable amounts of minerals, especially potassium, followed by calcium, manganese, sodium, and iron [27]. Nevertheless, the nutrient composition can vary substantially between members of the Apiaceae family, depending on their genetic structure, soil type, and location; this can also vary depending on the plant part analyzed [28]. Therefore, measuring the nutritional composition of different parts and species of fennel and dill is of great significance in the view of the comprehensive utilization of such plants.

### 3.3. Comparison of EO Yields from Different Samples

**Figure 3:**
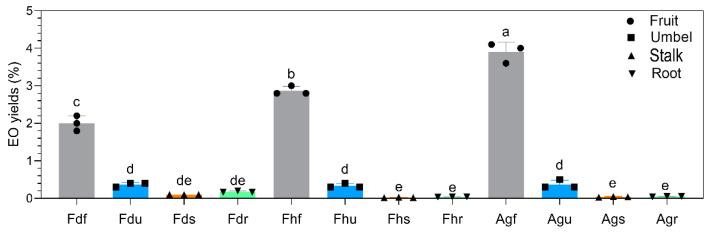
The evaluation of EO yields of all samples. Different letters indicate significant differences (*p* < 0.05).

EOs can be extracted from all parts of Fd, Fh, and Ag. The evaluation of EO yields, shown in Figure 3, indicated significant differences (*p* < 0.05) between the varieties and parts. These values ranged from 0.04–3.90% *v*/*w* (dry matter). The lowest EO yields were obtained in the stalks and roots (<0.2% *v*/*w*), and the highest EO yield was obtained in the fruits (≥2% *v*/*w*). The EO yields of fruits from Fd, Fh, and Ag were 2% *v*/*w*, 2.9% *v*/*w*, and 3.9% *v*/*w*, respectively. The EO yield of Fhf was similar to that of a previous study about fennel fruit (~3% *v*/*w*) [29]. Meanwhile, the EO yield of Agf was higher than a previous study about dill fruit by different hydrodistillation techniques (<4% *v*/*w*) [30]. Umbels (Fdu, Fhu, and Agu) across all the samples provided intermediate EO yields (~0.35%). Interestingly, the EO yield of Fd fruits was lower than that of Fh and Ag, but the EO yield of the stalks and roots of Fd was higher than that of Fh and Ag.

In the Apiaceae family, the accumulation of EOs is delimited to specialized structures located along with the vegetative and reproductive organs of plants, known as secretory ducts (or oil ducts) and vittae, respectively [31]. In the present study, the fruits provided the highest EO yield, which might be explained by the storage of oil in the oil ducts in fruits, as shown in Figure 1. In the EO extraction process, as the oil duct structures ruptured, the surface area and permeability of cells increased after grinding. This was conducive to the extraction of EOs and increased the EO yields [32]. Different ecological functions may be reflected in the EO content differences between different plant parts. In general, Apiaceae fruits, which contain reproductive organs, are optimum sources of biologically active compounds, regardless of the recovery rate or relative amounts [2]. The substantial difference in the EO yields between fennel and dill fruits could be explained by the “allocation strategy.” Dill “invests” more than fennel in the accumulation of dry matter in biomass and fruits, stretching the yield potential of each individual plant to the maximum level [33]. In addition, the content and quality of Apiaceae EOs are closely related to season, harvest time, climate, soil, and irrigation [34]. For instance, the optimal harvest time for dill in terms of maximizing the EO yield is at dawn, when a plant is fully growing, and the flower buds are fully open [8]. This coincides with the harvest time (at dawn) of this study.

### 3.4. Comparison of EO Constituents

**Figure 4:**
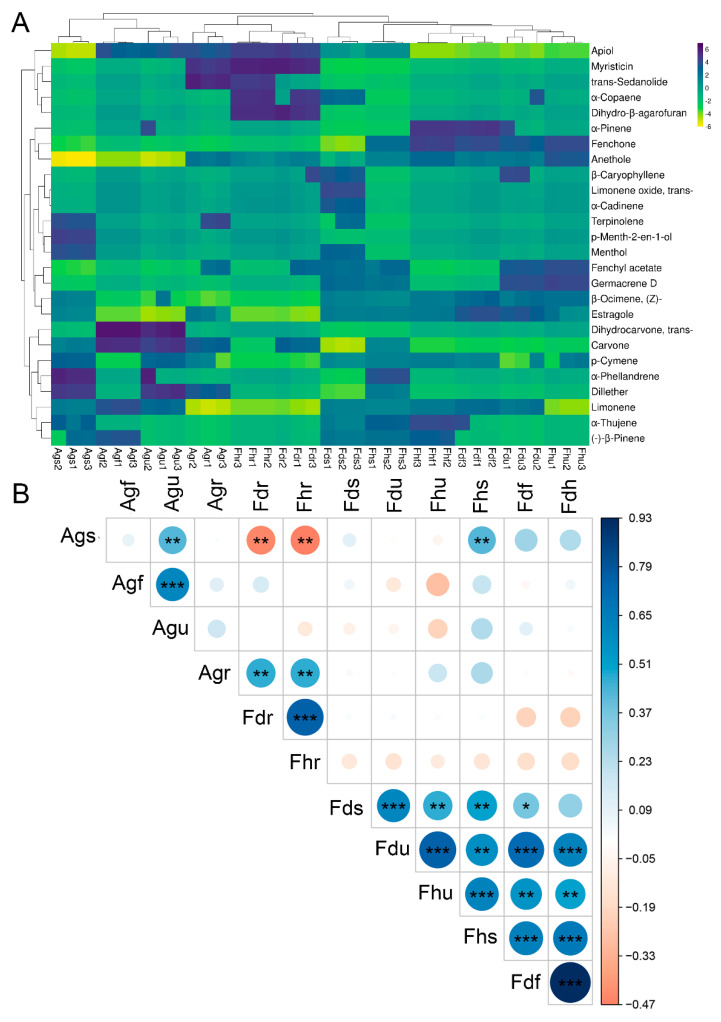
Heatmap (**A**) and Spearman rank correlation plot (**B**) based on EO chemical profiling. (* *p* < 0.05, ** *p* < 0.01, *** *p* < 0.001).

In this study, twenty-six compounds were identified, accounting for 97.71–99.63% of the total EO components, and the composition of the EOs varied greatly among parts and species. Heatmap analysis further visualized the differences in detail (Table 1; Figure 4A). Our results showed that two or three components of EOs exhibited relatively high proportions, and the remaining components usually existed at trace levels. Estragole was the dominant component (>80%) in the fruits of Fdf and Fdu. Anethole was the dominant component (>70%) in the EOs of Fhf and Fhu. Estragole and anethole have also been reported as the main components in the EOs of fennel fruit; their average composition was 34.90 and 56.81%, respectively [35]. Agf EOs contained the highest levels of limonene (50.19%) and carvone (42.41%). Karimi et al. [36] previously reported that limonene and carvone in the EOs of dill seed occupied 3.66 and 26.48%, respectively. Carvone and apiole in the EOs of Agu accounted for 45.78 and 23.83% of the total EO components, respectively. Distinctively, apiole was the main component in the roots of Fd, Fh, and Ag, accounting for 92.85, 94.24, and 67.13%, respectively. The contents of dill ether, α-phellandrene, and p-cymene in Ags EOs accounted for 28.50, 28.44, and 25.67%, respectively. The previous study reported leaves and stems from fennel having higher α-phellandrene content (>15%), while the levels were minimal in the seeds [37]. This was similar to the current results, where α-phellandrene was only detected in Fhs EOs (stems) from fennel samples. Interestingly, the content of α-phellandrene of EOs from Ags in this work was also found to be highest among all samples. Overall, estragole, anethole, apiole, carvone, and limonene were the main chemical components of Fd, Fh, and Ag. In terms of sample correlations, significant positive correlations were observed between Fdf and Fhf, followed by Fdu and Fhu, Fdr and Fhr, Fdu and Fdf, and Agf and Agu (Figure 4B). These samples have significant similarities in terms of chemical composition. Conversely, there were some negative correlations between samples, such as Fdr and Ags, Fhr and Ags. The results suggest that our samples can be distinguished based on their chemical composition.

**Figure 5:**
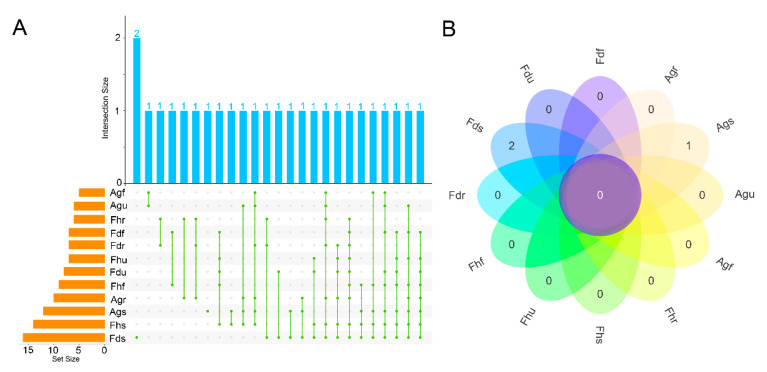
UpSet plot (**A**) and Venn diagram (**B**) based on the amounts of chemical components in EOs.

Moreover, an UpSet plot was obtained to visualize the distributions of shared and unique components of different parts and species (Figure 5A). The results revealed that the number of components present in each EO type ranged from 5 to 16. Of these, the majority of components were shared among multiple species. For instance, anethole was present in all fennel samples, and similarly, estragole was found in all fennel samples, except for the root samples. Carvone was present in different parts of Ag, and its content was higher in Agf and Agu than in Ags and Agr. Myristicin was unique to the roots of Fh, Fd, and Ag. Interestingly, the EO yield of the stalks was low; however, more components were identified in the stalks than in the other parts. Three components, limonene oxide (Fds), trans-α-cadinene (Fds), and p-menth-2-en-1-ol (Ags), were unique to specific species (Figure 5B). The aforenoted results provide new insights on the shared and unique components among different plant species and plant parts.

### 3.5. Multivariate Statistical Analysis of EO Chemical Profiles

**Figure 6:**
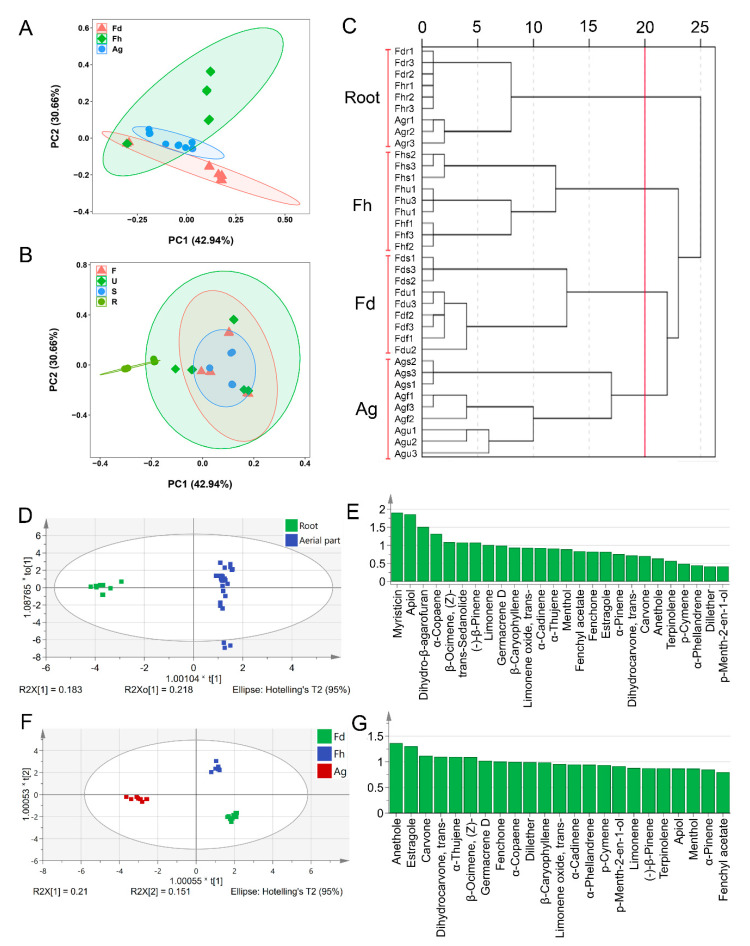
PCA map (**A**,**B**), dendrogram (**C**), score plots (**D**), and VIP values (**E**) from OPLS-DA analysis based on the chemical profiles of roots and aerial parts. Score plots (**F**) and VIP values (**G**) from OPLS-DA analysis based on the chemical profiles of three species (except for the roots).

To further classify the EOs in the fruits, umbels, stalks, and roots from the three species (Fd, Fh, and Ag), two PCA plots were constructed based on the species and different parts, respectively (Figure 6A,B). Overall, an improved classification was obtained based on the species than that based on the parts, except for the roots. Similarly, dendrogram analysis of EO chemical profiles yielded four main groups. Notably, the roots of different species were clustered into one group, and the samples of other parts were clustered into three other groups based on the species (Figure 6C). These results indicate that the components in the roots of the three cultivars exhibited more similar trends than those in the other parts, whereas the aerial parts (fruits, umbels, and stalks) of a specific species exhibited a similar EO composition. The EO chemical profiles of the roots were significantly different from those of the aerial parts. This may be because the roots were buried in the soil, leading to a significantly different growth environment from that of the aerial parts. The chemical compositions of EOs in the fruits, umbels, and stalks of a species are more similar than those in the same plant parts of different species, which might be explained based on the genotype. Yaldiz and Camlica [38] evaluated the chemical composition, mineral content, and antioxidant effect of 46 types of fennel plants and found significant differences between different fennel genotypes.

According to the dendrogram analysis results (Figure 6C), a supervised OPLS-DA statistical method was further used to distinguish the EO components between roots and aerial parts (Figure 6D,E) and among the aerial parts of the different species, Fd, Fh, and Ag (Figure 6F,G). The variable importance in projection (VIP) values of the PLS-DA model were used to screen chemical markers and to determine the contribution of chemical components to the PLS-DA model [39]. Figure 6E shows the VIP values of each EO component, and the vital components were identified (VIP value ≥ 1). Of these, myristicin (1.90399) and apiol (1.85922) significantly contributed to the classification via the OPLS-DA model; thus, these may be appropriate chemical markers to distinguish the EOs from the root and aerial parts of fennel or dill. As reported previously, myristicin and apiol are typical components of EOs isolated from Apiaceae roots [2], while anethole (1.36571), estragole (1.30292), and carvone (1.11947) may be appropriate chemical markers for distinguishing the EOs of aerial parts from Fd, Fh, and Ag.

In addition, EO chemical profiles may vary substantially between parts of the same plant species [40]. In particular, the composition of EOs varied between the root and aerial parts of Fd, Fh, and Ag. Interestingly, the EO chemical profile of the Apiaceae family is particularly different from that of the aerial parts and roots of *Cuminum cyminum* L. [41], *Daucus muricatus* L. [42], and *Thapsia villosa* L. [43]. Notably, 115 compounds were found in the aerial parts of *Carpesium cernuum* EOs, whereas only 37 compounds were found in the roots, which is consistent with previous studies. The proportions of the main compounds also vary greatly; for example, α-pinene (35%) and 2,5-dimethoxy-p-cymene (12%) were the predominant compounds in the EOs of shoots, whereas 2,5-dimethoxy-p-cymene (55%) and thymol isobutyrate (9%) were predominant in the EOs of roots [44].

## 4. Conclusions

In conclusion, this study provides fundamental information regarding the nutritional composition and EO profiles of fennel and dill. Multivariate statistical analysis showed that EOs extracted from root parts were significantly different from those obtained from the other parts. Some potential chemical markers (i.e., myristicin and apiol) were identified for distinguishing the arterial parts (fruits, umbels, and stalks) and roots of fennel and dill. Anethole, estragole, and carvone were identified as vital chemical markers for differentiating the plant species Fa, Fd, and Ag. These results provide a new understanding of the chemical characteristics of different parts of fennel and dill, which is significant for the efficient utilization of fennel and dill as food additives. In the future, to fully utilize the medical and nutritional properties of fennel and dill, the determination of specific chemical compositions from different parts and species is essential. Accordingly, we suggest that fruits are a good source of EOs, and also can be a source of further compounds with food additive relevance, of which the biological activity is worth further exploration. Umbels act as an important by-product of fennel and dill, whose EOs and minerals should be efficiently utilized and worth exploring. Furthermore, stalks have a large biomass and can be used as feed additives, whose nutritional compositions should be further explored. Taken together, the consumption of umbels and stalks would also reduce the amount of waste produced annually from the global food industry.

## Figures and Tables

**Table 1:** Chemical compositions of essential oils derived from different parts of fennel and dill.

Compound	RI	Fdf	Fdu	Fds	Fdr	Fhf	Fhu	Fhs	Fhr	Agf	Agu	Ags	Agr
α-Thujene	929	0.18 ± 0.31 ^cd^	-	0.1 ± 0.05 ^d^	-	1.86 ± 0.18 ^b^	-	2.39 ± 0.43 ^a^	-	-	-	0.56 ± 0.44 ^c^	-
α-Pinene		0.56 ± 0.1 ^b^	0.06 ± 0.1 ^c^	-	-	1.64 ± 0.05 ^a^	-	-	-	-	0.06 ± 0.1 ^c^	-	-
(−)-β-Pinene	990	-	-	0.26 ± 0 ^a^	-	0.27 ± 0.04 ^a^	-	0.35 ± 0.09 ^a^	-	0.07 ± 0.01 ^b^	-	0.15 ± 0.14 ^b^	-
Fenchone	1086	1.72 ± 0.17 ^c^	0.88 ± 0.14 ^d^	-	-	16.74 ± 0.61 ^a^	1.08 ± 0.18 ^d^	2.13 ± 0.06 ^b^	-	-	-	-	-
α-Phellandrene	1001	-	-	-	-	-	-	2.14 ± 0.49 ^b^	-	-	3.87 ± 6.7 ^b^	28.44 ± 0.05 ^a^	-
p-Cymene	1022	0.05 ± 0 ^d^	0.04 ± 0.07 ^d^	1.1 ± 0.02 ^c^	-	0.28 ± 0.01 ^d^	0.07 ± 0.08 ^d^	2.25 ± 0.05 ^b^	-	-	2.12 ± 0.87 ^b^	25.67 ± 0.23 ^a^	0.14 ± 0.12 ^d^
Limonene		7.88 ± 2.13 ^bcd^	7.98 ± 7.9 ^bcd^	41.77 ± 0.55 ^a^	-	3.91 ± 0.24 ^cd^	-	12.58 ± 0.49 ^b^	-	50.19 ± 7.71 ^a^	15.53 ± 9.17 ^b^	10.39 ± 0.21 ^bc^	-
β-Ocimene, (Z)-	1039	0.8 ± 0.4 ^b^	0.49 ± 0.49 ^bc^	2.26 ± 0.31 ^a^	-	0.2 ± 0.02 ^cde^	0.15 ± 0.02 ^cde^	0.41 ± 0.09 ^bcde^	-	-	0.06 ± 0.1 ^de^	0.43 ± 0.01 ^bcd^	-
Dihydrocarvone, trans-	1201	-	-	-	-	-	-	-	-	2.2 ± 0.29 ^b^	3.06 ± 0.78 ^a^	-	-
Terpinolene	1088	-	-	0.09 ± 0.08 ^b^	-	-	-	-	-	-	-	0.26 ± 0.01 ^a^	0.16 ± 0.14 ^b^
p-Menth-2-en-1-ol	1139	-	-	-	-	-	-	-	-	-	-	0.42 ± 0.07 ^a^	-
Limonene oxide, trans-	1137	-	-	1.05 ± 0.06 ^a^	-	-	-	-	-	-	-	-	-
Menthol	1173	-	-	0.16 ± 0.02 ^b^	-	-	-	-	-	-	-	0.21 ± 0.05 ^a^	-
Dill ether	1183	-	-	-	-	-	-	0.1 ± 0.02 ^c^	-	-	5.32 ± 0.42 ^b^	28.5 ± 0.19 ^a^	0.34 ± 0.02 ^c^
Estragole	1220	85.56 ± 2.27 ^a^	81.58 ± 6.74 ^a^	45.25 ± 0.46 ^b^	-	3.33 ± 0.13 ^d^	0.42 ± 0.64 ^d^	24.62 ± 0.61 ^c^	-	-	-	2.29 ± 0.11 ^d^	2.5 ± 2.13 ^d^
Fenchyl acetate	1219	-	0.98 ± 0.09 ^b^	3.83 ± 0.62 ^a^	0.09 ± 0.08 ^b^	-	0.79 ± 0.11 ^b^	3.3 ± 2.46 ^a^	-	-	-	-	0.12 ± 0.11 ^b^
Carvone	1246	-	-	-	0.31 ± 0.05 ^c^	-	-	0.32 ± 0.04 ^c^	-	42.41 ± 2.92 ^b^	45.78 ± 4.7 ^a^	0.38 ± 0.23 ^c^	2.72 ± 0.29 ^c^
Anethole	1286	2.79 ± 0.53 ^f^	6.8 ± 1.94 ^e^	0.69 ± 0.8 ^fg^	1.24 ± 0.92 ^fg^	71.17 ± 1.08 ^b^	96.8 ± 0.37 ^a^	46.79 ± 1.68 ^c^	0.24 ± 0.17 ^g^	-	-	-	16.86 ± 1.95 ^d^
α-Copaene		-	0.03 ± 0.06 ^c^	0.31 ± 0.01 ^b^	0.45 ± 0.39 ^ab^	-	-	-	0.67 ± 0.04 ^a^	-	-	-	-
β-Caryophyllene	1420	-	0.04 ± 0.04 ^b^	0.49 ± 0.02 ^a^	0.02 ± 0.03 ^b^	-	-	-	-	-	-	-	-
Germacrene D	1484	-	0.41 ± 0.05 ^b^	0.73 ± 0.02 ^a^	-	-	0.3 ± 0.05 ^c^	0.15 ± 0.02 ^d^	-	-	-	-	-
Dihydro-β-agarofuran	1490	-	-	-	0.34 ± 0.03 ^a^	-	-	-	0.28 ± 0.01 ^b^	-	-	-	-
Apiol	1640	-	-	0.69 ± 0.51 ^d^	92.85 ± 1.77 ^a^	-	-	1.35 ± 0.18 ^d^	94.24 ± 0.87 ^a^	4.63 ± 4.85 ^d^	23.83 ± 9.65 ^c^	-	67.13 ± 0.49 ^b^
Myristicin		-	-	-	3.67 ± 0.91 ^a^	-	-	-	4.07 ± 0.85 ^a^	-	-	-	4.38 ± 0.12 ^a^
α-Cadinene		-	-	0.13 ± 0.03 ^a^	-	-	-	-	-	-	-	-	-
trans-Sedanolide		-	-	-	-	-	-	-	0.1 ± 0.01 ^b^	-	-	-	3.4 ± 0.09 ^a^
Total		99.54 ± 0.05	99.29 ± 0.29	98.92 ± 0.09	98.96 ± 0.51	99.39 ± 0.06	99.61 ± 0.12	98.87 ± 0.34	99.6 ± 0.04	99.49 ± 0.31	99.63 ± 0.34	97.71 ± 0.66	97.75 ± 0.44

Notes: ‘-’ means not detected; data are mean ± standard deviation (SD); Means with different letters in a row are statistically significant (*p* < 0.05).

## Data Availability

Not applicable.
